# Optimizing campus-wide COVID-19 test notifications with interpretable wastewater time-series features using machine learning models

**DOI:** 10.1038/s41598-023-47859-2

**Published:** 2023-11-24

**Authors:** Tuo Lin, Smruthi Karthikeyan, Alysson Satterlund, Robert Schooley, Rob Knight, Victor De Gruttola, Natasha Martin, Jingjing Zou

**Affiliations:** 1 Department of Biostatistics, University of Florida, Gainesville, FL 32608 USA; 2 Division of Engineering and Applied Science, California Institute of Technology, Pasadena, CA 91125 USA; 3 Student Affairs, University of California, San Diego, La Jolla, CA 92093 USA; 4 Division of Infectious Diseases and Global Public Health, Department of Medicine, University of California, San Diego, La Jolla, CA 92093 USA; 5 Department of Pediatrics, University of California San Diego, La Jolla, CA 92093 USA; 6 Department of Computer Science and Engineering, University of California, San Diego, CA USA; 7 Center for Microbiome Innovation, University of California, San Diego, CA USA; 8 Herbert Wertheim School of Public Health and Human Longevity Science, University of California, San Diego, La Jolla, CA 92093 USA

**Keywords:** Public health, Statistics

## Abstract

During the COVID-19 pandemic, wastewater surveillance of the SARS CoV-2 virus has been demonstrated to be effective for population surveillance at the county level down to the building level. At the University of California, San Diego, daily high-resolution wastewater surveillance conducted at the building level is being used to identify potential undiagnosed infections and trigger notification of residents and responsive testing, but the optimal determinants for notifications are unknown. To fill this gap, we propose a pipeline for data processing and identifying features of a series of wastewater test results that can predict the presence of COVID-19 in residences associated with the test sites. Using time series of wastewater results and individual testing results during periods of routine asymptomatic testing among UCSD students from 11/2020 to 11/2021, we develop hierarchical classification/decision tree models to select the most informative wastewater features (patterns of results) which predict individual infections. We find that the best predictor of positive individual level tests in residence buildings is whether or not the wastewater samples were positive in at least 3 of the past 7 days. We also demonstrate that the tree models outperform a wide range of other statistical and machine models in predicting the individual COVID-19 infections while preserving interpretability. Results of this study have been used to refine campus-wide guidelines and email notification systems to alert residents of potential infections.

## Introduction

The ongoing spread of SARS CoV-2 creates an urgent need for rapid detection of the SARS CoV-2 virus that aids in development of effective decision making to contain its transmission in communities—particularly those with high density congregate living such as university campuses^1–3^. Campus-wide surveillance systems capable of rapid detection of new infections remain an important public health priority^4–7^.

Wastewater surveillance has been demonstrated to be a cost-effective approach to monitoring viral spread, by virtue of its ability to (1) detect individual infections at early stages in some settings, (2) identify variants of concern, and (3) provide a less biased assessment of population infection dynamics–particularly in settings where infections are underreported to health departments^8–16,19,20^.

As part of the “Return to Learn” (RTL) program of the University of California, San Diego (UCSD), a campus-wide GIS (geographic information systems)-enabled wastewater surveillance system has been implemented for the detection of SARS CoV-2 since Fall 2020^17,18,34^. Currently, the program has 131 samplers collecting daily from > 340 buildings (both residential and non-residential). A previous study at UCSD from 2020 showed that the wastewater surveillance system was highly sensitive in detecting individual infections (85% of the buildings where a residential student was diagnosed with SARs-COV-2 had a positive wastewater signal prior to individual identification). Additionally, notification of building residents that their building had a positive signal doubled testing rates among residents, even during a period of routine asymptomatic testing^19^. Information on wastewater results is provided on the UCSD public daily dashboard, and targeted email notifications are sent to those living or working in buildings with concerning signals.

A key question challenging programs using wastewater for early detection is when targeted notifications, including email notifications, should be issued to populations at risk in order to increase testing or enhance other mitigation efforts to contain potential transmissions. Crucial to answering this question is quantitative assessment of the relationship between the risk of individual COVID-19 infections and the wastewater test results from associated samplers. There is a recognized need for real-time analysis of the wastewater results to inform decision making ^56^.

Results from correlative studies have demonstrated a significant relationship between the viral load in wastewater and individual COVID-19 PCR-based test results. Vallejo et al.^21^ used a linear model for the relationship between COVID test cases and viral load detected in the wastewater in A Coruña, Spain. Bar-Or et al.^22^ also applied a linear model and concluded that the concentration of the virus RNA in the Bnei Brak sewage correlates with the number of COVID-19 positive individuals in the city. Agrawal et al.^23^ found a significant correlation between COVID-19 incidence and viral load observed in wastewater in the Frankfurt metropolitan area. Li et al.^24^ performed a meta-analysis for multi-national wastewater data and compared three different models, multiple linear regression, artificial neural network, and adaptive neuro fuzzy inference system for predicting COVID-19 community prevalence (# of infections per 100,000 people) based on wastewater-based quantities including the SARS-CoV-2 RNA concentration.

Several studies utilized not only wastewater results from single time points but also longitudinal time series of wastewater data. Krivoňáková et al.^25^ found a high correlation between the number of viral particles in wastewater and the number of individual cases tested 2 weeks later in data from Bratislava. Cao et al.^26^ analyzed the time series of wastewater results using the vector autoregression model to model the weekly variations on the SARS-CoV-2 wastewater concentrations and COVID-19 cases in the Borough of Indiana, PA. Ai et al.^27^ compared different time-series and non-time-series machine learning and deep learning models including linear model, gradient-boosting decision tree, feed-forward deep neural networks, Facebook Prophet and long short-term memory for the predictive performance of COVID-19 cases in central Ohio. Their results indicated that time-series models outperformed non-time-series models. Other studies^28–30^ have also compared advanced neural networks to predict COVID-19 cases. However, few existing studies focused on extracting interpretable predicting features from time series wastewater results and using them to predict individual test results, which is crucial for facilitating transparent and informed community-level decision making as well as evaluations of the reliability and robustness of the decisions. Comparing to black-box type models, models that can identify the importance of features are particularly advantageous because they provide decision makers with a clear understanding of the factors that contribute to the model's predictions, allowing for more targeted interventions and informed decision making.

In this study, we propose a new pipeline for feature extraction of longitudinal wastewater-based testing results and predicting individual COVID-19 infections with the features. As we discuss below, wastewater testing is one example of pooled testing^31–33^. What is different in our setting is that in standard pooling, investigators can control and standardize how many samples are pooled and how much sample from each person is contributed. In our setting, these factors are impacted by the design of wastewater systems and depend on processes that experimenters do not control. But some principles remain the same; and our analyses are examples of evaluation of diagnostic tests—in our case wastewater tests—based on their properties: sensitivity, specificity, positive and negative predictive values. Wastewater test results are used to predict the outcome at the level of sets of residence buildings that are associated with manholes in which samplers have been installed. The outcome we seek to predict is whether or not at least one person is infected in the set of buildings associated with a given sampler. We use machine learning to make use of longitudinal time series of wastewater tests to develop optimal rules for notification based on the test properties.

Specifically, we develop hierarchical classification/decision tree models to select important features from the longitudinal series of tests that should trigger notification—that is, that makes it likely that at least one resident is positive. Our analyses of the data on wastewater tests and infections among residents at UCSD derive from information collected in the period from Nov. 2020 to Nov. 2021, covering approximately a whole academic year. Results indicate that by leveraging single-day, long-term and short-term features extracted from the time series of wastewater results, the classification tree model can predict the presence of a positive resident with high sensitivity and satisfactory specificity. Important wastewater features are identified in a hierarchical manner; the most important feature is having a positive wastewater test in at least 3 out of 7 past days. If fewer than 3 out of 7 past days have positive wastewater test results, then the next most important feature is whether 1 out of 5 past days have positive wastewater tests. When applying the model to a set-apart testing set, the prediction accuracy is 72.3%. We also compare the performance of the proposed model to that of random forest models as a benchmark and other commonly used statistical and machine learning models; results indicate the proposed model can predict outcomes with equal or better accuracy while maintaining a high level of interpretability.

Findings derived from the proposed approach have been used to evaluate and refine the current notification system at UCSD. This system sends out timely email notifications to alert residents to a positive wastewater sample associated with their residence buildings and recommend individual COVID-19 tests to contain transmissions at early stages^19^. As a result of this study, in 2021 UCSD modified the email notification system to notify after 3 days of a positive signal. However, during the Omicron surge the email notifications were issued after 2 positive days due to the short viral kinetics, indicating the need for ongoing analysis as the virus and epidemiology change.

Our study addresses the urgent need for real-time analysis of data from wastewater surveillance systems and predictive models using wastewater features to predict COVID-19 infections. Results of our study facilitate informed decision making for community-level recommendations and policies intended to contain and prevent transmissions of COVID-19. The approach proposed here provides accurate prediction of individual COVID-19 infection and interpretable feature engineering and can be readily implemented and applied to other similar systems.

## Data processing and model

### Pre-processing of wastewater test results

**Figure 1:**
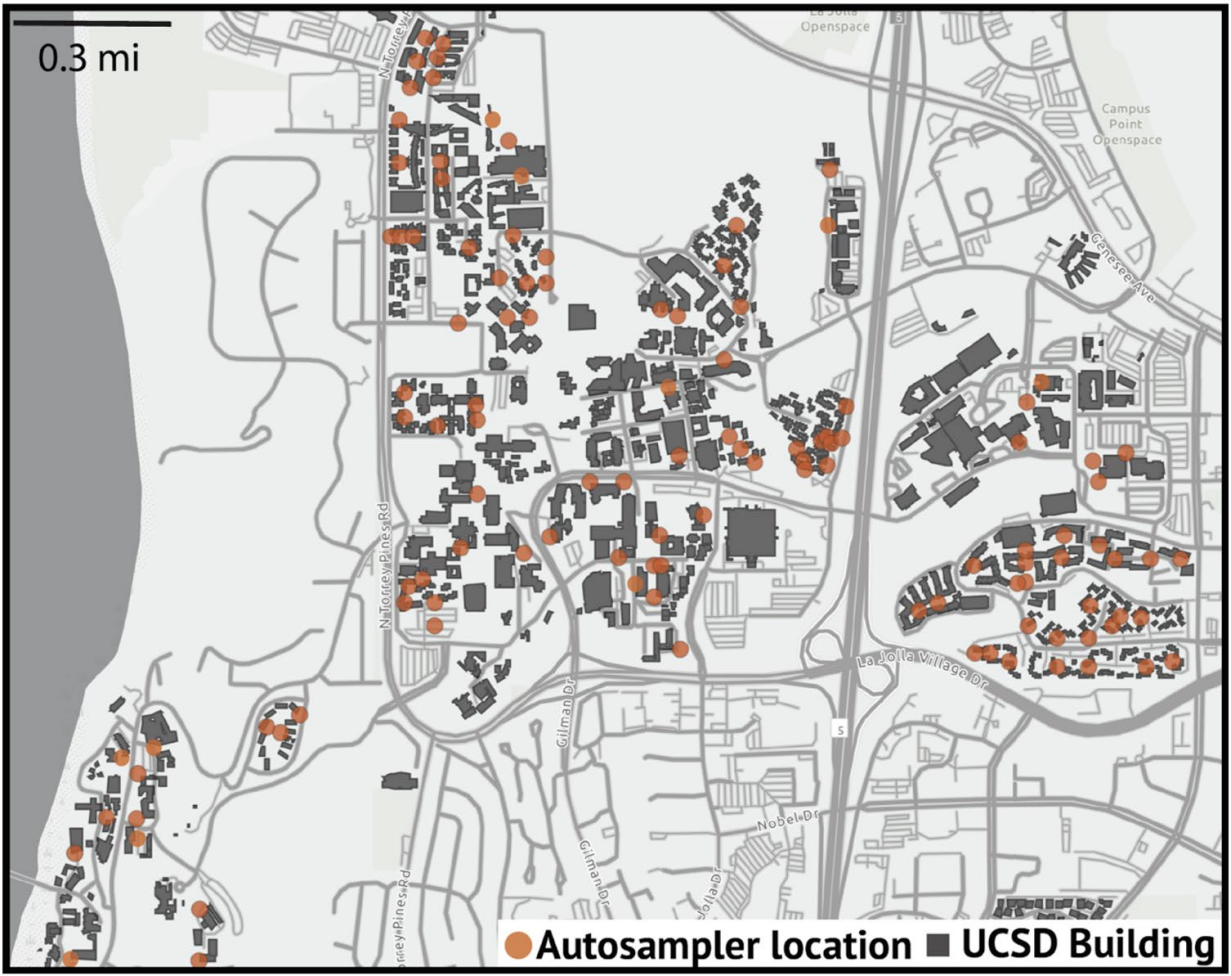
Locations of autosamplers installed in manholes (orange circles) connected to UCSD buildings (grey blocks)^20^.

As part of the UCSD return-to-learn program, a total number of 140 commercial auto-samplers have been deployed in manholes across the UCSD campus, covering teaching, administrative, and residence buildings, including four isolation buildings for students who test positive for COVID-19. In this study, we focus on the data from the 73 manholes covering the 239 residence buildings and their ~ 9700 residents. Figure 1^20^ shows the structure of manholes associated with residence buildings. Twenty-four-hour composite wastewater samples are collected daily from the manholes and analyzed in the laboratory for viral concentration. SARS-CoV-2 signatures are screened via real-time quantitative PCR (RT-qPCR) for the N1, N2, and the E genes^8^. Results are integrated with the campus GIS database to traceback from the manholes to associated upstream residence buildings and identify potential sources of any positive SARS-CoV-2 signals.

As mentioned above, wastewater tests are used to estimate the sensitivity and specificity of different rules for predicting that at least one person will test positive among residents in a set of buildings associated with a given sampler. This requires tracing the source of positive signals back to buildings in a way that accounts for the upstream/downstream structure of the sewer network: only the buildings that can contribute to the wastewater are matched to a given manhole. Shown in Fig. 1 is the structure of manholes connecting to residence buildings^20^. However, the set of buildings associated with a sampler can depend on the results of the wastewater tests. For example, if wastewater from sampler B tests positive but that from an upstream sampler A tests negative, only the buildings contributing wastewater into the sewer between samplers A and B are considered relevant for analysis of signals in sampler B. By contrast, if both samplers are positive, then all buildings associated with either A or B are included in the analysis. The spatially enabled sewer network and subsequent trace of samplers to buildings were stored in and performed by ArcGIS Pro 2.7 (Esri). More details about the sewer network and tracing of samplers can be found in^19^ and the interactive web interface at https://returntolearn.ucsd.edu/dashboard/index.html.

Our analysis focuses on the time period of 11/23/20–11/13/21, which covers the majority of the academic year 2020–2021 and the first quarter of year 2021–2022. A total of 23,282 wastewater daily samples were collected during this period, and a cutoff of the quantification cycle [Cq] values 39^8,20^ was used to categorize these samples as positive (< 39) vs. negative (> = 39). Among the samples, 3488 were positive and 19,794 were negative.

### Ascertainment of individual tests results of COVID-19

During the COVID-19 pandemic, UCSD student residents were required to take individual COVID-19 tests weekly (reduced to bi-weekly after Spring 2021). In addition, in an effort to alert individuals of potential infections in their buildings and encourage them to be tested in one of the on-campus diagnostic testing sites or self-administered test-kit vending machines, targeted email notifications were sent to residents of associated source buildings when positive wastewater SARS-CoV-2 signals were detected in manholes. Notices were also sent to the UCSD campus when a potentially positive building contained a common access area open to the public^19^. Tests are sent to UC San Diego Health labs for processing and the results are saved in an electronic health record (EHR) system^17,34^. Results of individual tests are available within one day of testing.

Daily individual diagnostic COVID-19 test results of residents in each building are aggregated and merged with the daily wastewater results from manholes associated with the buildings. After excluding all the missing observations, there are a total of 8853 daily wastewater test records in the merged data, of which 1212 are positive and 7641 are negative. The corresponding COVID-19 individual diagnostic test results among students residing in campus housing indicate 170 are positive and 8683 are negative.

Of the 170 COVID-19 individual diagnostic positive test results among students residing in campus housing, only 54.7% have a tested-positive wastewater sample from the associated manhole on the same day of the individual test, indicating using daily wastewater test results alone cannot achieve satisfactory prediction of individual infections of COVID-19 in associated buildings. Potential reasons for the observed discrepancy between individual tests and wastewater results include delays in being tested or getting results among those who had become infected. For example, among infected residents, there could be a delay in the manifestation of symptoms or absence of symptoms; for those reasons or others, the individual tests may not take place until a few days after the actual onset of the infection. There can also be false negative wastewater test results arising from low viral concentration, even if one or more residents in associated buildings have become infected. In addition, there is a possibility of false positives in the wastewater results. To understand the implications of the wastewater samples and to optimize the utility of the wastewater surveillance system in detecting individual infections, a definition of the outcome of individual infections that accounts for potential lags between the wastewater and individual test results is needed.

Here we propose a 3-day time window approach to define the outcome of individual infections. Using the date of wastewater test as an anchor point, for each manhole we examine individual diagnostic test results of residents in associated buildings in the 3-day window including the date of wastewater test and the day before and after the wastewater test. This outcome is defined as positive for an individual-level test if there exists at least one positive individual COVID-19 test result among residents in associated buildings in this time window. The proposed time window addresses the time lag between the wastewater and individual tests by including positive individual tests in intervals of one day before to one day after the detection of a positive wastewater test. A sensitivity analysis using a longer window of 6 days has also been conducted and its results are described in the Appendix; this choice of window leads to a similar model as does the analysis with a 3-day window.

### Model for predicting individual COVID-19 infections using wastewater results

To detect individual COVID-19 infections, we use multiple interpretable features extracted from wastewater time series data, which include both single-day test results and short-term/long-term trends, to provide a comprehensive characterization of different aspects of the wastewater test results. The features integrated in the model are: (1) a list of features includes single-day wastewater results up to five days before the day in question, (2) counts of positive signals among the past days including whether at least 1 out of the past 3 days, 1, (or 2, 3) out of the past 4 days, 1 (or 2, 3) out of the past 5 days and 2 (or 3) out of the past 7 days contains positive wastewater signals, and (3) features characterizing trends in the past days such as whether wastewater results are all positive in the past 3 consecutive days.

We adopt a machine learning approach–classification trees–^35–37,42,57^, to predict individual COVID-19 infections defined using the 3-day window with the above features extracted from wastewater signals. The classification tree derives from a hierarchical model that predicts outcomes with recursive binary partitions based on an ordering of the importance of the predictors. At each node/leaf of the classification tree, the feature capable of reducing the maximal amount of Gini impurity, a criterion to measure the mixture of different classes of the outcome, is selected to partition the data^38–40^. Predictors that appear in earlier nodes are considered more important in predicting the outcome^42,57^. The ordering of importance of predictors is crucial in our study, as we aim to accurately predict the presence of infections in residence buildings and to reveal important and interpretable features from wastewater test results to aid in decision making for campus-wide recommendations and mandates. To avoid overfitting and improve interpretability, we apply constraints on the model complexity using a penalty parameter *cp*^41,43,44,57^. In addition, the classification tree mitigates collinearity among predictors as a result of its variable selection mechanism based on feature importance^50^.

We also incorporate a re-weighting mechanism in our model to address the important issue of imbalance in the outcome. There are many more negative than positive individual test results in the data, which represents a typical imbalance in the outcome of individual testing of COVID-19 in similar communities. Models optimizing prediction accuracy when trained with the data without any adjustment tend to classify all outcomes as negative due to over-representation of the negative outcomes. To address this issue, we re-weight the data by allocating larger weights to positive than to negative outcomes in training the classification tree models. This approach is similar to over-sampling the minority class and under-sampling the majority class, which has been shown to achieve good classifier performance^45–47^.

To evaluate the performance of the proposed approach, we partition the data from 11/23/20 to 11/13/21 into a training and a testing set. The training set includes data from 11/23/20 to 04/30/21 and the testing set includes data from 06/30/21 to 11/13/21. The partition of the dataset is not random: it preserves the chronological ordering of the dates of the test results as definitions of the features extracted from the wastewater samples rely on the chronological ordering of the dates. In addition, results in the same period are expected to behave similarly as the policies, circulating variants, and other pandemic conditions vary with the chronologic time of measurement. Comparing model performance in the training and testing sets also provides insight into the influence of these factors on the effectiveness of the wastewater surveillance system. We exclude the samples in May and June due to potential data quality issues; further investigation of the wastewater results during this period is needed. In the Appendix, we present a sensitivity analysis that includes data from this period, and we obtain the same model as described in the following section. This analysis serves to demonstrate the robustness of our results.

## Results

### Classification tree trained with the training set

**Figure 2:**
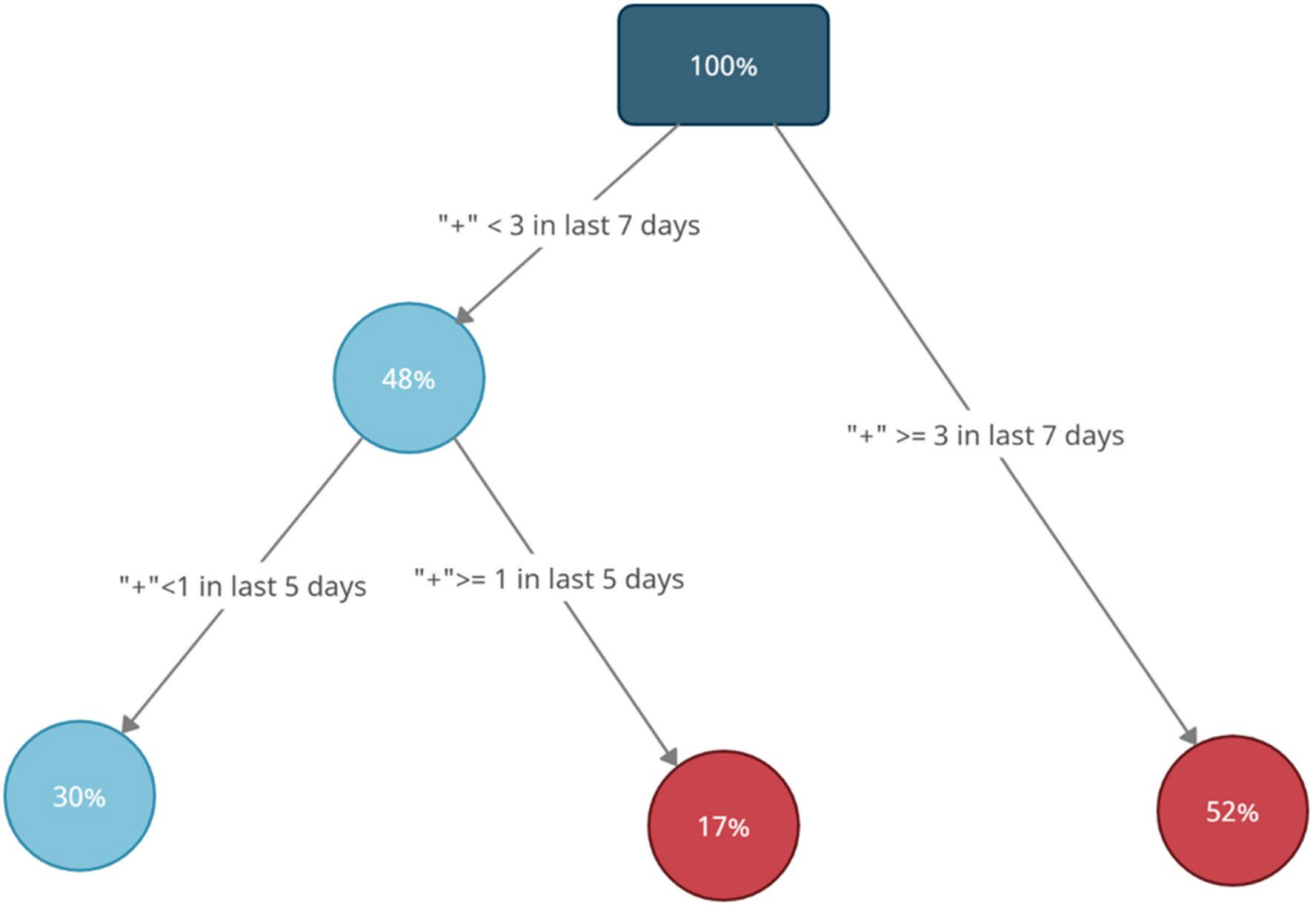
Classification tree model trained with the training set only. Wastewater time series features are used to predict individual COVID-19 test results. The red node means a positive predicted outcome and the blue node means a negative predicted outcome. The value inside each node denotes the percentage of the total data records that falls in the category of the node. “+” means number of positive wastewater results. For example: “ +  < 3 in last 7 days” means there were less than ( <) 3 days of positive wastewater results in the last 7 days of wastewater testing.

Figure 2 shows the result of classification tree trained with the training set. From the top (root) to the bottom (leaves) of the tree, we show the features selected to predict the outcome; features closer to the root are considered to be more important. The branches of each node, visualized by the arrows, describe the features and the two possible conditions used for binary partitioning of the data according to which condition is satisfied. The color of each node indicates the predicted outcome for records partitioned into the category corresponding to the node: red indicates a positive predicted outcome of at least one infection in associated buildings, and blue, a negative predicted outcome. The value in the circle of each node indicates the percentage of the partitioned data records in the whole data.

The model in Fig. 2 indicates the most important feature in predicting the outcome is whether fewer than (<) 3 days in the last 7 had positive wastewater test results. The outcome is predicted to be positive if wastewater results are positive in at least 3 out of the past 7 days, and negative otherwise. Given positive wastewater results on fewer than 3 out of the past 7 days, the second most important feature is whether none of the past 5 days have positive wastewater results. If yes then the outcome is predicted to be negative, otherwise to be positive.

The classification/decision tree in Fig. 2 is fitted with weights of positive outcomes equal to (2/# positive classes) and weights of negative outcomes equal to (1/number # of negative classes).

Note the weights are standardized by the total number of positive and negative outcomes, respectively, and then multiplied by scalers based on the importance placed on correctly predicting the positive and negative outcomes. Our choice of weights reflects the priority of sensitivity (true positive rate) over specificity (true negative rate) in predicting positive individual infections. A sensitivity analysis using weights equal to the reciprocal of class sizes for both classes is performed in Appendix. The value of the penalty parameter on model complexity *cp* = 0.02 is chosen to balance optimal performance in the training set as suggested by cross-validation while maintaining a small number of nodes in the tree for model interpretability. A sensitivity analysis using *cp* = 0.001 to train the model is available in the Appendix to further investigate the influence of model complexity on the prediction performance and the trade-off between model complexity and interpretability.

Table 1 shows the confusion matrix of the predictions when applying the model to the training set. The sensitivity (True Positive Rate, TPR = TP/(TP + FN)) is 83.7% and the specificity (True Negative Rate, TNR = TN/(TN + FP)) is 58.5%. Note that the calculations of sensitivity and specificity are unaffected by the weights allocated to positive and negative outcome classes as the weights appear in both numerators and denominators and cancel out. The overall weighted prediction accuracy is 75.3%, which is calculated by

$$\frac{{\mathop \sum \nolimits_{i = 1}^{n} w_{i} \left[ {I\left( {predict{ }\;positive{|}positive} \right) + { }I\left( {predict\;{ }negative{|}negative} \right)} \right]{ }}}{{\mathop \sum \nolimits_{i = 1}^{n} w_{i} }}$$

where $w_{i}$ denotes the weight of sample $i$, $I\left( {predict{ }\;positive{|}positive} \right)$ denotes the indicator function that sample $i$ has a positive outcome that is predicted to be positive, and $I\left( {predict{ }\;negative{|}negative} \right)$ denotes the indicator function of sample $i$ has a negative outcome that is predicted to be negative. It is expected to observe a higher estimated sensitivity than specificity as we are over-sampling the positive outcome class compared to the negative class.

**Table 1:** Confusion matrix of results obtained from applying the model (trained with the training set) to the training set.

	Predict positive (%)	Predict negative (%)
Actual positive	83.7	16.3
Actual negative	41.5	58.5

To evaluate the prediction performance of the classification tree, we then apply the model to the set-apart testing set in the period of 06/30/21–11/13/21. The confusion matrix is provided in Table 2. For the testing set, the sensitivity decreased from 83.7 to 77.1% while the specificity increased from 58.5 to 62.8%. The overall weighted prediction accuracy is 72.3%. The testing set contains the period in which most of the student residents had already received vaccination and the wave of the highly infectious SARS-CoV-2 Omicron variant had not yet arrived^49^. Therefore, fewer infected cases were observed and thus underrepresented the total population. Despite the evolving nature of the pandemic, the model performed well and was able to predict individual infections with satisfactory accuracy and high sensitivity. We also trained a model on the testing set alone and compared it with the model trained with the training set; the comparison of results is available in the Appendix.

**Table 2:** Confusion matrix of results obtained from applying the model (trained with training set only) to the testing set.

	Predict positive (%)	Predict negative (%)
Actual positive	77.1	22.9
Actual negative	37.2	62.8

### Influence of weights

In this section we investigate the role of relative weights of positive and negative outcomes in the prediction. For simplicity of notation, we denote a relative weight of (*a*/#positive classes): (*b*/#negative classes) for positive vs. negative outcomes as *a:b*. For example, the model in Fig. 2 is trained with weights 2:1; this weighting places a double amount of emphasis on records with positive outcomes compared to those with negative outcomes after standardizing by the total numbers of positive and negative outcomes. The trained classification tree model for relative weights 1:1 is available in the Appendix as a sensitivity analysis.

**Figure 3:**
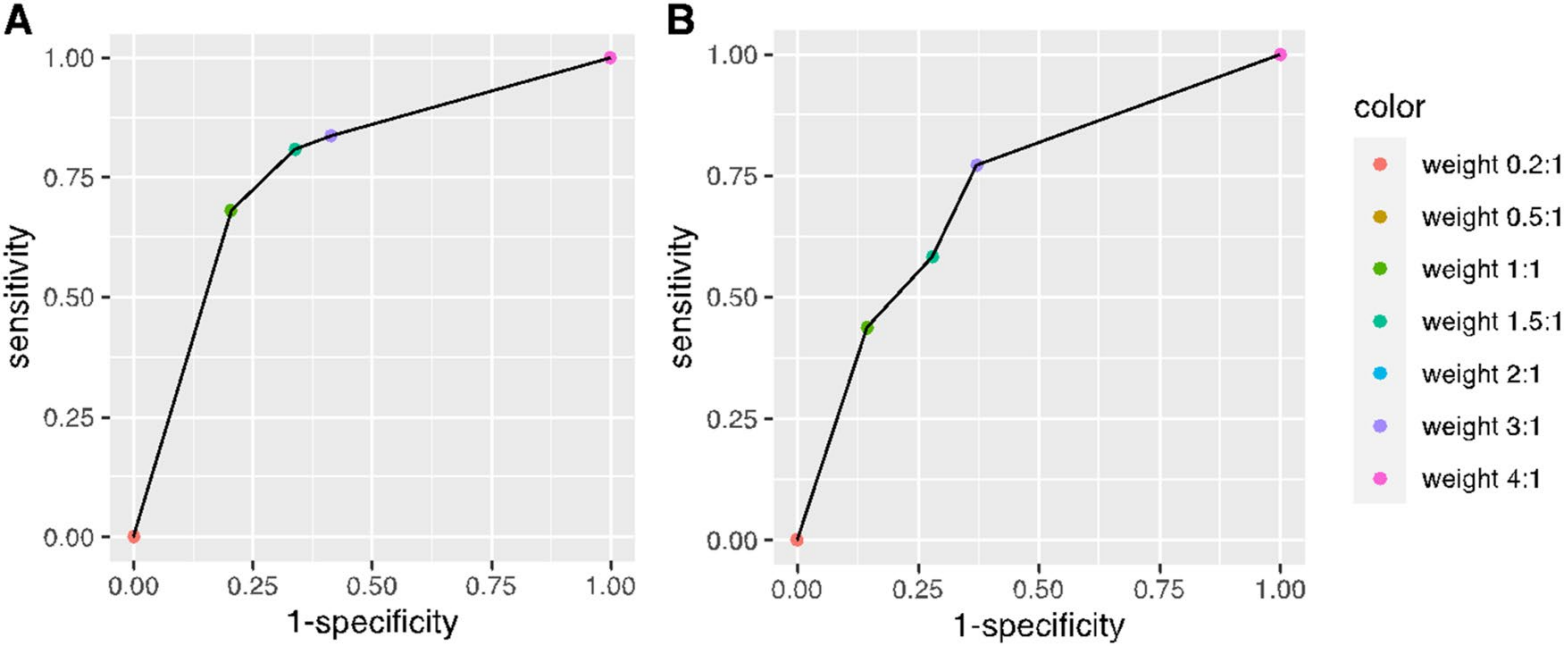
ROC (Receiver Operating Characteristic) curves of models trained with different relative weights for positive and negative outcome classes using data of the training set only. The left panel shows results obtained from applying the models to the training data. The right panel shows results of applying the models trained with the training set to the testing set.

Figure 3 displays the receiver operating characteristics (ROC) curve^48,51^, which demonstrates a trade-off between sensitivity and specificity; the *x*-axis indicates one minus the specificity, and the *y*-axis indicates the sensitivity. This curve permits a comparison of the performance of models trained with varying weights. Detailed results are provided in Table 3. With relative weights on the positive class as small as 0.2:1, all the outcomes are predicted to be negative; hence, the sensitivity is 0 and the specificity is 1. As the weight for positive class increases, the sensitivity also increases, and the specificity decreases. With relative weights of 4:1 or greater, all outcomes are predicted to be positive, yielding sensitivity of 1 and specificity of 0.

**Table 3:** Detailed values of sensitivity and (1−Specificity) for ROC curves in Fig. 3.

Relative weight (positive vs. negative outcome)	Sensitivity (training set performance) (%)	1−Specificity (training set performance) (%)	Sensitivity (testing set performance) (%)	1−Specificity (testing set performance) (%)
0.2:1	0	0	0	0
0.5:1	68.1	20.4	43.8	14.5
1:1	68.1	20.4	43.8	14.5
1.5:1	80.7	33.8	58.3	28.0
2:1	83.7	41.5	77.1	37.2
3:1	83.7	41.5	77.1	37.2
4:1	100	100	100	100

Table 4 summarizes the importance of features in models trained with different weights given by orders of nodes appearing in the classification trees. For results to be comparable, *cp* value of 0.02 is used in training all models with different weights; this approach leads to different numbers of nodes under different weight settings. For all models, the root nodes are defined by whether or not fewer than 3 out of the past 7 days have positive wastewater signals; this is consistently the most predictive wastewater feature for predicting individual COVID-19 infections. In all models with a lower level node/leaf, the next most important feature is whether or not none of the previous 5 days have positive wastewater signals. Combined with the result of the root node, a predictive model that is robust to the choice of weights consistently includes the dichotomous features: 3 or more out of 7 days wastewater positive (yes/no) and 1 to 5 of the previous days wastewater positive (vs 0 days). This model leverages features characterizing wastewater results both in a long-term trend of 7 days and in shorter periods of 5 days.

**Table 4:** Importance of features extracted from wastewater time series given by models trained with different relative weights.

Relative weights	0.5:1	1:1	1.5:1	2:1	3:1
1st level feature	3_out_7	3_out_7	3_out_7	3_out_7	3_out_7
2nd level feature			1_out_5	1_out_5	1_out_5
3rd level feature			2_out_7		

“*a*_out_*b*” in the table represents the dichotomous feature of whether there were at least *a* out of the previous *b* days with positive wastewater test results.

### Prediction with random forest model as a benchmark

To further evaluate the prediction performance of the proposed classification tree model, we apply a weighted random forest model^52^ consisting of an ensemble of 1000 individual weighted classification trees. As in the classification tree model, weights are applied for oversampling the positive individual cases. The random forest is known for its high prediction accuracy but lacks the interpretability of the classification trees. Comparing the performance of the proposed model to that of the random forest enables us to assess the proposed model with a reliable benchmark and to understand the trade-off between the interpretability and prediction accuracy of models.

Detailed results are provided in Tables 5 and 6. The proposed classification tree models generally outperform the random forest models in the same weight settings, especially when the relative weights of positive vs. negative outcomes are high. For the random forest approach, a choice of weight ratio that leads to high sensitivity and relatively high specificity, is 3:1. In this case, sensitivity equals to 72.9% and specificity equals to 68.5%, leading to a 71.7% prediction accuracy, while the proposed classification tree model has a prediction accuracy of 73.5% (at the same 3:1 weight ratio). One possible reason for the random forest to under-perform compared to the proposed classification tree is that the random forest is based on bootstrap (or subsampling) of the data, which breaks the chronological structure of the time series in the data and thereby potentially affects the prediction performance. Another possible reason is that given the relatively small feature space and the limited number of positive COVID infections in the data, the increased complexity of the random forest model introduces more risk of overfitting, which likely contributed to its decreased accuracy when applied to unseen test data. Furthermore, the random forest model is not the preferred choice in our study due to its reduced interpretability and transparency, particularly for the purpose of guiding campus-wide policies.

**Table 5:** TPR (Sensitivity) and FPR (1−Specificity) for different machine learning models under different relative weights, calculated from applying models trained from the training set to the testing set.

Relative weights	Classification tree		Logistic regression		Logistic regression (LASSO)		SVM		Neural network		Random forests	
	TPR	FPR	TPR	FPR	TPR	FPR	TPR	FPR	TPR	FPR	TPR	FPR
0.2:1	0	0	4.2	0.4	0	0	0	0	0	0	0	0
0.5:1	43.8	14.5	29.2	10.1	39.6	10.3	37.5	8.8	37.5	9.2	35.4	9.2
1:1	43.8	14.5	54.2	20.2	43.8	14.5	54.2	18.0	47.9	16.1	52.1	13.9
1.5:1	58.3	28.0	64.6	26.6	77.1	37.2	68.8	29.4	64.6	28.2	64.6	25.6
2:1	77.1	37.2	66.7	30.2	77.1	37.2	72.9	32.6	70.8	32.5	70.8	30.6
3:1	77.1	37.2	75.0	34.4	77.1	37.2	100	100	66.7	29.6	72.9	31.5
4:1	100	100	97.9	97.7	100	100	100	100	87.5	92.6	95.8	95.5

The (%) sign is omitted for space saving.

**Table 6:** Weighted prediction accuracy for different machine learning models under different relative weights, calculated from applying models trained from the training set to the testing set.

Relative weights	Classification tree (%)	Logistic regression (%)	Logistic regression (LASSO) (%)	SVM (%)	Neural network (%)	Random forests (%)
0.2:1	83.3	83.7	83.3	83.3	82.9	83.3
0.5:1	71.6	69.7	73.0	73.3	74.6	72.4
1:1	64.6	67.0	64.6	68.1	67.5	68.8
1.5:1	63.8	68.1	71.4	69.5	68.1	68.4
2:1	72.3	67.7	72.3	71.1	69.0	70.3
3:1	73.5	72.7	73.5	75.0	67.6	71.7
4:1	80.0	78.8	80.0	80.0	71.7	77.7

### Comparisons to other statistical and machine learning models

Besides the random forest models, we also assess the proposed classification tree model against various commonly used statistical and machine learning models, thoroughly evaluating their predictive performance and interpretability. All of these models are fitted using identical features extracted from the wastewater signals, same training and testing data partitioning, and the same weight ratios of positive vs. negative outcomes as for the classification tree models, ensuring a fair comparison. Results listed in this section focus on the model performance under the weight ratio of 2:1 as in the proposed classification tree model. Complete results under a variety of weight ratios can be found in Tables 5 and 6.

First, we apply both the logistic regression model and the logistic regression with LASSO regularization^53^ for variable selection to our preprocessed data. The ten-fold cross-validation is used to determine the value of the penalty parameter lambda for LASSO. The threshold of 0.5 for the predicted probability of positive individual infection is used to determine the binary predicted outcome. Logistic regression without variable selection produce a relatively low prediction accuracy of 67.7% with sensitivity of 66.7% and specificity of 69.8% when applying the model fitted using the training set to the set-aside test set. The observed under-performance of prediction in the test set may be due to its higher model complexity, which can lead to overfitting. Logistic regression with LASSO yields improved accuracy of 72.3% with sensitivity of 77.1% and specificity of 62.8% in the test set. Variables selected using LASSO include indicators of positive wastewater signals in at least 3 days out of past 7 days, at least 1 day out of past 5 days and at least 1 day out of past 3 days, largely overlapping with the important features selected by the classification tree model and hence the similar results. Although LASSO selects one additional variable, compared to the decision tree method, it has a very similar prediction performance (exactly the same in 3 decimal digits). This is because the variable whether wastewater signals are positive in at least 1 day out of past 3 days has a regression coefficient very close to 0 (despite not exactly equal to 0). As a popular variable selection method, LASSO is considered a comparable approach to the decision tree in our study, but it is less intuitive in terms of ranking the variable importance in prediction, which is a critical factor we consider in our policy making process.

We also apply several machine learning models including the Support Vector Machine (SVM) with linear kernel and Feedforward Neural Network (FNN) with single hidden layer^54,55^. Both traditional SVM and FNN do not support variable selection and have limited interpretability. Furthermore, the prediction performance of these two methods falls short of the classification tree method in the test set (SVM: accuracy: 71.1%, sensitivity: 72.9%, specificity: 67.4%; FNN: accuracy: 69.0%, sensitivity: 70.8%, specificity: 67.5%). Notably, FNN exhibits impressive performance in the training set, achieving an accuracy of 78.1%. This underscores that complex machine learning methods can excel at fitting the training data but may encounter overfitting issues when applied to unseen testing dataset. Furthermore, when the weight ratio of positive vs. negative increases to 3:1, the SVM loses its effectiveness, resulting in a specificity of 0 and predicting all outcomes as positive.

Tables 5 and 6 include detailed results of sensitivity vs. (1-specificity) when applying training-set-fitted models with different weight ratios to the test data, using each of the models in comparison. The classification tree methods and logistic regression with LASSO are the two approaches that strike a good balance between interpretability and high sensitivity, particularly when using weight ratios of 2:1 and/or 3:1. Overall, the proposed classification tree model still possesses the best prediction accuracy. Given its good prediction performance and interpretability of results, the logistic regression with LASSO can serve as a viable alternative to the classification tree model. Nevertheless, from the perspective of policy makers, the classification tree may still hold an advantage due to its intuitive feature importance ranking. Further details on prediction accuracy, sensitivity and specificity for training models can be found in the Appendix.

### Positive predictive value (PPV) and negative predictive value (NPV)

We further examine the positive predictive value (PPV) and negative predictive value (NPV) of the predictions of individual infections as defined below:

$$\begin{aligned} {\text{Positive}} & \, \;{\text{predictive}}\;{\text{ value }}\left( {{\text{PPV}}} \right) \, \;{\text{of}}\;{\text{ wastewater}}\; \, \left( {{\text{WW}}} \right)\;{\text{ test}} \; \\ \, & \; = \frac{{{\text{Sensitivity}}\;{\text{ of }}\;{\text{WW}}\;{\text{ test }}*{\text{ prevalence}}}}{{\left\{ {\left( {{\text{sensitivity }}*{\text{ prevalence}}} \right) \, + \, \left( {{1} - {\text{specificity}}} \right) \, \left( {{1 }{-}{\text{ prevalence}}} \right)} \right\}}} \\ & \; = \, {\text{ TP}}/\left( {{\text{TP}} + {\text{FP}}} \right) \\ \end{aligned}$$

$$\begin{aligned} {\text{Negative}} & \;{\text{ predictive}}\;{\text{ value }}\;\left( {{\text{NPV}}} \right) \, \;{\text{of}}\;{\text{ WW}} \;{\text{ test}} \\ & \;\; \, = \frac{{ {\text{Specificity }}\;{\text{of }}\;{\text{WW}}\;{\text{ test }}* \, \left( {{1} - {\text{prevalence}}} \right) }}{{\left\{ {{\text{specificity }}*\left( {{1} - {\text{ prevalence}}} \right) \, + \, \left( {{1} - {\text{ sensitivity}}} \right) \, \left( {{\text{prevalence}}} \right)} \right\}}} \\ & \;\; = {\text{ TN}}/\left( {{\text{TN }} + {\text{ FN}}} \right) \\ \end{aligned}$$

where TP and FP are numbers of true and false positives and TN and FN are numbers of true and false negatives in the prediction, and the prevalence is the proportion of true positives among all tested units of observation (which could be, for example, at a building or individual level).

These quantities can be particularly useful in developing policies regarding control of the COVID-19 epidemic. In the case of pooled tests, results can help in using testing resources more efficiently—by focusing intensive testing where cases are most likely to reside. In addition, the tests can provide an early warning about the potential for at least one resident of a building unit to be infected. To make best use of the wastewater tests, we estimate the probability that there is at least one infected person in a residence given a positive wastewater test. This estimate will aid in evaluating the cost–benefit of different strategies for testing the residents. In addition, knowledge of the relationship between the timing of positive wastewater tests and positive individual-level tests can inform us about when—or at what schedule—it is best to offer the latter to residents.

Our testing setting is a little more complex than usual, because the wastewater test is a pooled test that aggregates results of buildings associated with the same manholes; hence, the number who contribute to the pool varies across tests—which are done at the residence level. Furthermore, the prevalence of interest is at the residence level; as noted above, we define a residence to be a true positive if there is at least 1 infected resident in the residence. Like the wastewater itself, this definition is at the residence building level.

The prevalence at the residence building level *p*_*c*_ can be estimated from the prevalence *p* at the individual level given the number of residents (*n*), under the assumption of independence across infection events across them: *p*_*c*_ = *prob of (*> = *1 infected resident)* = *1−(1−p)*^*n*^ where *p* is individual-level prevalence. Because most detected infection events we observed are only in a single person, we believe that violation of this assumption has little effect on our estimates. As the prevalence of COVID-19 and the number of residents vary with date, the estimates of PPV and NPV will vary with date as well. There are also possible dilution effects that could affect the estimations. For example, the detectability of SARS CoV-2 genetic material may depend on the total number of residents living in the upstream of the manholes.

**Figure 4:**
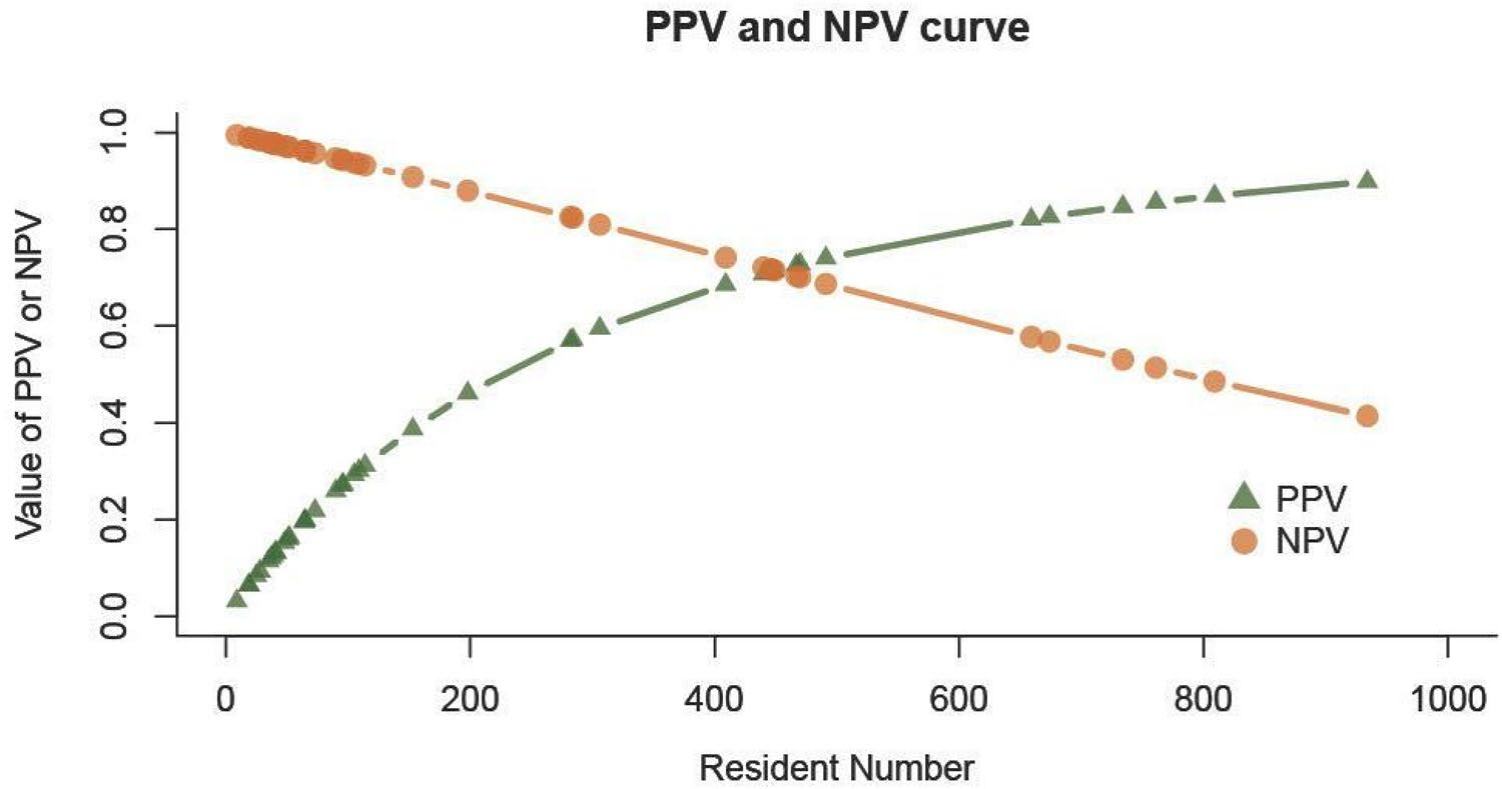
PPV and NPV curves as functions of numbers of residents in buildings associated with manholes.

Here we provide approximate building-level estimates of the PPV and NPV and demonstrate how they are affected by the number of residents in buildings associated with manholes. We focus on the period of the week before Fall 2021 quarter begins, as most student residents are in the process of moving back onto campus during that week, and are required to take individual-level tests as soon as they move into their residences. The curves of PPV and NPV as a function of the number of people in residence buildings are shown in Fig. 4. We note that the PPV and NPV are quite sensitive to the number of residents; the usefulness of wastewater tests must be considered in this context. Negative tests are less reassuring as the number climbs near 1000; whereas PPV only approaches 50% when the number of residents is near 250.

### Sensitivity analysis

Furthermore, we conduct a comprehensive sensitivity analysis designed to assess the model's performance. We systematically vary sampling settings and model parameters and compare the proposed approach to other models and methods and evaluate the results. Specifically, the sensitivity analysis includes: (1) altering the time-window length in defining the outcome of individual COVID-19 infection, (2) applying different weight ratios of positive vs. negative outcomes in fitting the models, (3) varying model complexity including number of predictive features selected, (4) fitting a separate classification tree model using only the test set, (5) examining data from May and June 2021, (6) training proposed model on data including only Fall 2020, when the vaccines are still not publicly available, (7) varying the sampling frequency of wastewater signals, and (8) conducting a comparative analysis of the proposed classification tree model against other statistical and machine learning models. Details of the sensitivity analysis and results are available in the Appendix. Based on results from the above sensitivity analyses, we conclude that the proposed model and method stand as the overall best choice in the context of for our study. When applying the model, we recommend that researchers leverage our model for their own studies and carry out a similar sensitivity analysis to refine the parameter settings tailored to the specifics of their individual models.

## Concluding remarks

This paper proposes an innovative approach for predicting the presence of infections in residence buildings using results from wastewater surveillance systems. The goal of this study is to make use of wastewater test results to inform decision making regarding notification of wastewater results to guide public health strategies intended to control the spread of individual COVID-19 infections in communities. To this end, we extract features that characterize wastewater test results over time, develop classification/decision tree models to select important features, use them to predict probabilities that there is at least one individual infection in residences, and finally optimize the COVID-19 test notification strategy.

We used the classification tree to analyze data from the wastewater surveillance system and individual-level COVID-19 tests of residents on UCSD campus from Nov 2020 to Nov 2021. Results reveal that the best predictor of positive individual level tests in residence buildings is whether or not the wastewater results were positive in at least 3 of the past 7 days. Using a set-apart testing set, we demonstrate the accuracy of these predictions. Our results suggest that the proposed analysis approach can be useful in using wastewater to guide policies around notifications for building residents to seek individual-level testing. Features included in the model are robust to changes in weights of positive and negative individual test results, and the features discovered to be most important are consistent across different weight settings in balancing the positive and negative outcomes in the data.

Our study contributes to the UCSD wastewater surveillance system by introducing a more streamlined and effective methodology for utilizing wastewater test data to inform campus-wide decision-making efforts aimed at reducing virus transmission and preventing outbreaks. We leverage advanced statistical and machine learning techniques to identify key features from time series of wastewater test results, optimizing the cost-effective utilization of the surveillance system's capabilities. Discoveries from the analysis have been useful in assisting decision making in the UCSD campus-wide Return-to-Learn program and have been incorporated into the UCSD email notification system.

Although our approach is motivated by and developed for the UCSD Return-to-Learn program, it can be readily applied to similar wastewater surveillance systems to predict individual COVID-19 infections in communities and to facilitate decision making processes in making community-wide guidelines, mandates and policies for containing transmission of the virus. In applying the proposed approach, several aspects of the model may need to be adjusted by researchers and/or policymakers according to pandemic conditions at the time of analysis. Detailed discussions regarding the potential limitations of the proposed approach when applied to other scenarios are presented in the following section.

## Discussion

This study has potential limitations which may affect the effectiveness of the proposed approach when applied in other scenarios. Here we discuss each of the limitations and provide directions to possible solutions. First, in defining the outcome of individual COVID-19 infections, we introduced a time window of 3 days to account for potential lags from onset of infections to testing and mismatches between the individual infections and the wastewater results. If the required test frequency changes, the optimal performance of wastewater tests may require that the time window be adjusted accordingly.

Second, conditions of the pandemic vary over time because of the regular appearance of new variants and changes in people’s behavior as responding to masking and other mandates and mitigation strategies. Furthermore, coverage rates of vaccinations may improve over time in some communities, but the effectiveness of older vaccines constantly wanes. Such external factors can influence the effectiveness of the model using wastewater test results. A possible solution to ensure the model reflects and adapts to these changing factors is to use the online learning approach and continuously update the model training as new data becomes available over time. Sensitivity analysis can also be conducted to examine the influence and importance of these factors.

Third, an important consideration is the trade-off between the cost and benefit of different wastewater testing strategies, which may vary in different applications. In our study, we utilized all available daily wastewater data. However, in some other scenarios, collecting daily wastewater signals can be costly. One possible solution is to adjust the data collection frequency to strike a balance between cost and benefit. We have presented a sensitivity analysis in the Appendix to assess the impact of different sampling frequencies. However, it's important to note that when working with data sampled at a different frequency, adjustments must be made to the feature engineering methods, as features derived from daily wastewater measurements may not be applicable with less frequent data collection. The trade-off between the sampling cost and the prediction accuracy of the model should also be examined carefully.

Another aspect of the cost–benefit trade-off is the choice between wastewater epidemiology and individual clinical COVID tests. At UCSD, the test costs approximately the same for wastewater samples and clinical samples: though technical costs are higher for wastewater due to an additional concentration step, labor costs are higher for clinical due to the need for licensed personnel, resulting in a rough balance. The samplers were roughly evenly distributed between residential and non-residential areas, with approximately 70 samplers serving 10,000 residents. When clinically testing 10,000 people twice a week, it results in 20,000 tests per week. In contrast, utilizing daily wastewater surveillance for a group of 70 individuals for a week totals 490 tests per week, illustrating substantial cost savings at this scale. Additionally, while one could argue that antigen tests are less expensive than qPCR, it's essential to consider the data capture costs, which are notably high. This is primarily because there is no automated method for individuals to report their test results, and they generally do not do so. The trade-off between cost and benefit can be its own topic and can be included as a potential future direction for research in this area. While we aim to provide some useful discussion here, we recommend that researchers conduct cost–benefit analyses and/or explore the long-term sustainability of wastewater surveillance for COVID-19 within the context of their own situation.

Finally, ethical and privacy concerns need to be addressed when applying the proposed approach to other scenarios. Ethical and privacy concerns are minimal in our application as wastewater was collected at the building level and does not contain identifiable information to trace back to specific individuals. Therefore, while the wastewater testing component helped improve the timeliness of email notifications, it did not introduce any additional data elements that could jeopardize privacy. However, these measures may not be universally applicable. Researchers seeking to replicate our approach should carefully consider the ethical implications specific to their context and adhere to relevant privacy regulations.

### Ethics declarations

The Institutional Review Board (IRB) of University of California, San Diego provided approval for human subject protection oversight of the data obtained by the EXCITE laboratory for the campus clinical samples. Informed consent was obtained from all participants included in the study, and the appropriate institutional forms have been archived, and any sample identifiers included were de-identified. The wastewater component of this project was discussed with our IRB and was not deemed to be human subject research as it did not record personally identifiable information. All methods were carried out in accordance with relevant guidelines and regulations.

### Supplementary Information

Supplementary Information.

## Data Availability

All raw wastewater sequencing data are available via the NCBI Sequence Read Archive under the BioProject ID PRJNA819090. Consensus sequences from clinical and wastewater surveillance are all available on GISAID. Spike-in sequencing data are available via Google cloud (https://console.cloud.google.com/storage/browser/search-reference_data). The UCSD campus dashboard can be accessed at https://returntolearn.ucsd.edu/dashboard/. The SEARCH genomic surveillance dashboard is available at https://searchcovid.info/dashboards/sequencing-statistics/. The wastewater time series features are available to researchers for non-commercial use per request.

## Code availability

The code for all analysis involved in this manuscript is hosted publicly on GitHub repository (https://github.com/tuolin123/Wastewater_UCSD).
