# Supplementary material for: Optimizing campus-wide COVID-19 test notifications with interpretable wastewater time-series features using machine learning models
Source: Sci Rep. 2023 Nov 24;13:20670. doi: 10.1038/s41598-023-47859-2 (PMC10673837; doi:10.1038/s41598-023-47859-2)
Supplement: Supplementary file 1 — Supplementary Information. [file 41598_2023_47859_MOESM1_ESM.docx]

**Supporting Information for**

Optimizing campus-wide COVID-19 test notifications with interpretable wastewater time-series features using machine learning models

Tuo Lin^a^, Smruthi Karthikeyan^b^, Alysson Satterlund^c^, Robert Schooley^d^, Rob Knight^e^, Victor De Gruttola^f^, Natasha Martin^d^, Jingjing Zou^g^*

^a^ Department of Biostatistics, University of Florida, Gainesville, FL, 32608, USA

^b^ Division of Engineering and Applied Science, California Institute of Technology, Pasadena, CA 91125, USA

^c^ Student affair, University of California, San Diego, La Jolla, CA, 92093, USA

^d^ Division of Infectious Diseases and Global Public Health, Department of Medicine, University of California, San Diego, La Jolla, CA, 92093, USA

^e^ Department of Pediatrics, University of California San Diego, La Jolla, CA, 92093, USA

Department of Computer Science and Engineering, University of California, San Diego, CA, USA

Center for Microbiome Innovation, University of California, San Diego, CA, USA

^f^ Department of Biostatistics, Harvard T.H. Chan School of Public Health, Boston, MA, 02115, USA

^g^ Division of Biostatistics and Bioinformatics, Herbert Wertheim School of Public Health and Human Longevity Science, University of California, San Diego, La Jolla, CA, 92093, USA

* Corresponding Author: Jingjing Zou, PhD, Assistant Professor, Division of Biostatistics and Bioinformatics, Herbert Wertheim School of Public Health and Human Longevity Science, University of California, San Diego, La Jolla, CA, 92093, USA. Phone: 917-226-5260.

**Email:**  j2zou@ucsd.edu

**This PDF file includes:**

Supporting text

Figures S1 to S7

Tables S1 to S13

**Appendix**

This appendix comprises a comprehensive sensitivity analysis to assess the proposed model's performance by systematically varying its parameters and comparing it to other models and methods, including (1) altering the time-window length in defining the outcome of individual Covid-19 infection, (2) applying different weight ratios of positive vs. negative outcomes in fitting the models, (3) varying model complexity including number of predictive features selected, (4) fitting a separate classification tree model using only the test set, (5) examining data from May and June 2021, (6) training proposed model on data including only Fall 2020, when the vaccines are still not publicly available, (7) varying the sampling frequency of wastewater signals, and (8) conducting a comparative analysis of the proposed decision tree model against other statistical and machine learning models. Based on results from the above sensitivity analyses, we conclude that the proposed model and method stand as the overall best choice in the context of for our study. When applying the model, we recommend that researchers leverage our model for their own studies and carry out a similar sensitivity analysis to refine the parameter settings tailored to the specifics of their individual models.

**Sensitivity Analysis**

**S1. Classification tree model trained with outcome defined with 6-day time window**

We perform a sensitivity analysis of defining the outcome using a 6-day time window, that is, buildings associated with a manhole on a specific date are considered to have positive individual infections if there is at least one positive result in the window starting from 2 days before to 3 days after the date in question. With the outcome defined by a longer time window, we expect to reduce bias caused by delays in individual COVID-19 tests. On the other hand, using a longer window in defining the outcome of individual test results in buildings could lead to potential mismatches between the actual dates of wastewater signals and individual infections. This sensitivity analysis provides an opportunity to examine further the trade-off between these biases.

Fig. S1 shows the classification tree model trained with the training set using the outcome defined by this 6-day time window and tree complexity cp = 0.02. Compared with the results using 3-day time window, this model has one more decision node, whether there are fewer than 2 out of the past 7 days with positive signals. The confusion matrix of this model in Tables S1 (training set) and S2 (testing set) exhibit similar sensitivity and specificity to the model trained with outcome defined by 3-day time window. The overall weighted prediction accuracy corresponds to Table S1 is 74% and corresponds to Table S2 is 70.7%.

**S2. Classification tree model trained with a different relative weight of positive vs. negative outcomes**

In the results section of the main text, we have analyzed the sensitivity and specificity of models with varying weights. Here we present additional results of the classification tree model trained with the training set using 1:1 relative weight in Figure S2. The *cp* value used in this model is 0.02. This model is simpler than the model trained using relative weight of 2:1: in the classification tree, there is only the root node of whether less than 3 out of past 7 days have positive signals.

The confusion matrices resulting from applying the training model to the training set and testing set are shown separately in Tables S3 and S4. These two tables show that with a 1:1 weight ratio, the sensitivity decreases while the specificity increases, especially when fitting the model to the testing set. The results confirm that the model trained with higher weights on positive outcome helps improve the sensitivity while lowering the specificity. The overall weighted prediction accuracy corresponds to Table S3 is 73.9% and is 64.6% corresponds to Table S4.

**S3. Adjusting tree complexity parameter *cp* for the training model**

In this sensitivity analysis, we use the parameter *cp* = 0.001, which is recommended by the cross-validation procedure, to train the classification tree models. Figure S3 visualize the model trained using this approach. The model has 19 nodes, which is considerably more complex than the one trained with *cp* = 0.02 in the main text, greatly reducing its interpretability.

When applying this model to the training set, the weighted prediction accuracy slightly increases to 78.2%, with higher sensitivity and specificity shown in Table S5. This is anticipated as the more complex model trained with the training set improves the model performance when applied to the same dataset. However, when applying this model to the set-apart test set, the prediction performance is worsened with prediction accuracy decreases to 66.5% and lower sensitivity and specificity as shown in Table S6. The reduced prediction accuracy in the test set suggests potential over-fitting when using a more complex model.

**S4. Fitting decision tree models on the test set only**

To explore further the characteristics of the testing dataset and whether there are significant changes in the data characteristics over time that require model adjustments to adapt to the changes, we conducted a sensitivity analysis. In this analysis, we fitted a separate classification tree model using only the test set. In this model, we continue to use the weight 2:1 and *cp* = 0.02 to be consistent with the model training in the main text. The fitted model is presented in Figure S4. The root node for this model is whether the day before had a positive wastewater test. If so, the outcome of the day in question will be predicted as positive. If the previous day does not show a positive wastewater test, then the next node of whether there were less than 2 out of past 7 days with positive signals. The remaining decision nodes include whether 3 days before (single-day) and less than 1 out of 5 past days had positive wastewater tests.

Compared with the model trained with the training set, this model trained using the testing set is more sensitive to recent wastewater test results, especially test result of the day before. As we discussed in the main text, the testing set data is from the time when most students are vaccinated and before the delta and omicron wave, and the testing policy also relaxed from weekly to biweekly to the time when wastewater signals appear for vaccinated students. Thus we expect to see that students will take the individual test immediately after they get notification of positive signals from wastewater results, with the results coming one day after. This indicates that our wastewater surveillance system is successful.

The confusion matrix of the model is provided in Table S7. The overall weighted prediction accuracy is 74.2%. Comparing to weighted prediction accuracy 72.3% when applying the model trained with the training set to the testing set, the improvement is marginal. This suggests that the model trained with only the training set performs well enough for satisfactory predictions.

**S5. Including the samples in May and June in the analysis**

In training the model in the main text, we excluded data from May and June 2021 due to data quality issues that require further investigation: summary statistics reveal that there were only 2.2% of positive wastewater samples in these two months, comparing to 13.5% in the training set, 14.2% in the testing set, and 16.9% of the period during the summer schools (July and August).

As a sensitivity analysis, here we include data from May and June 2021 in training set and re-train the model. Figure S5 shows the resulting classification tree. This model is in fact the same as that in the main text, while the sensitivity and specificity in the new training set as shown in Table S8 is different due to the additional data. The sensitivity decreases from 83.7% to 74.8% and the specificity increases from 58.5% to 72.9%, and the weighted prediction accuracy decreases from 75.3% to 74.2%. Since the models are the same, the results of applying this classification tree to the testing set (from period 06/30/21 - 11/13/21) remain the same.

**S6. Using the data** **only from Fall 2020 for the training set**

On December 14^th^, 2020, the first American outside of clinical trials received the COVID-19 vaccine [CDC 2023]. This was also the time when winter vacation started and many students traveled back home for Christmas and New Year events, with a higher risk of infection. In training the proposed model in the main paper, we incorporated data from this period, as well as data from the time when vaccines were publicly available, into the training set. The resulting training set preserves more heterogeneity from different periods. In this sensitivity analysis, we exclusively utilize data from Fall 2020 (from period 11/23/20 – 12/14/20) for the training set while maintaining the same testing set as in the previous analysis. This approach allows us to examine the impact of vaccination on the predictive performance of the model.

Figure S6 shows the resulting classification tree from fitting the new training data. The most important features are whether there are positive wastewater signals for at least 3 days out of past 7 days. Additionally, the other two features, namely, whether there were positive wastewater signals yesterday and for at least 1 day out of the past 5 days, are also selected in this tree.

Tables S9 and S10 show the confusion matrices of this model for the training and testing sets, with tree complexity cp = 0.01. The sensitivity are 94.9% and 56.3% and specificity are 45.3% and 68.8% in training and testing set, respectively. Notably, the accuracy drops from 78.3% in the training set to 60.4% in the testing set. This substantial decrease in performance can be attributed to the smaller sample size and homogeneity of data from Fall 2020 before vaccines were publicly available, which compromises the model's ability to make accurate predictions in a more heterogeneous testing set.

**S7. Adapting different sampling frequencies for wastewater data**

In some scenarios, daily wastewater monitoring may be costly. In this sensitivity analysis, we evaluate the trade-off between the cost of wastewater sampling and the effectiveness of using wastewater features to predict individual Covid-19 infections. We inspect the model performance under different sampling frequencies of the wastewater signals. In addition to the daily sampling frequency discussed in the main paper, we also evaluate the performance of the proposed model suppose wastewater signals are sampled at intervals of every 2 or 3 days, with wastewater results in between these intervals being treated as unobservable, and provide a comparative analysis of the results.

It is important to note that the frequency of wastewater sampling directly affects the availability of predictive features that can be derived. For instance, in the case of sampling every 3 days, not all of the daily wastewater features in the main paper (such as wastewater signals 1, 2, 4, 5 ...days preceding the current day) can be observed. Here we incorporate two predictors in the model: whether wastewater signals from 3 days before and whether those from 6 days before are positive. In the scenario of sampling wastewater signals every 2 days, daily wastewater features considered are whether there are positive signals 2, 4, or 6 days before the current day. Furthermore, we incorporate features that assess whether there are positive wastewater signals in 2 out of the past 4 days and 3 out of the past 6 days into the model.

In the case of sampling every 3 days, the model did not identify any predictors as predictive of the outcome, resulting in positive predictions for all subjects. For sampling every 2 days, the resulting tree model is shown in Figure S7, which includes all the single day predictors (whether 2, 4 or 6 days before contains positive wastewater signals). The training and testing confusion matrices are displayed in Tables S11 and S12. The sensitivity is 81.2% and 57.1% and specificity are 65.8% and 69.7% in training and testing set, respectively. The overall prediction accuracy drops from 76% in training to 61.3% in the test set, indicating a notably lower predictive performance due to the loss of information when wastewater signals are sampled less frequently.

**S8. Comparing the predictive performance with other machine learning methods**

We have also compared the proposed approach with other statistical and machine learning models in terms of predictive performance. In addition to the results presented in the main paper, we provide here additional results from this comparative analysis. Tables S13 provides details on the sensitivity, specificity, and accuracy of all other methods in the training sets (since testing sets performance has already been shown in the main text), by using the 2:1 weight ratio of positive vs. negative outcomes.

Fig. S1.
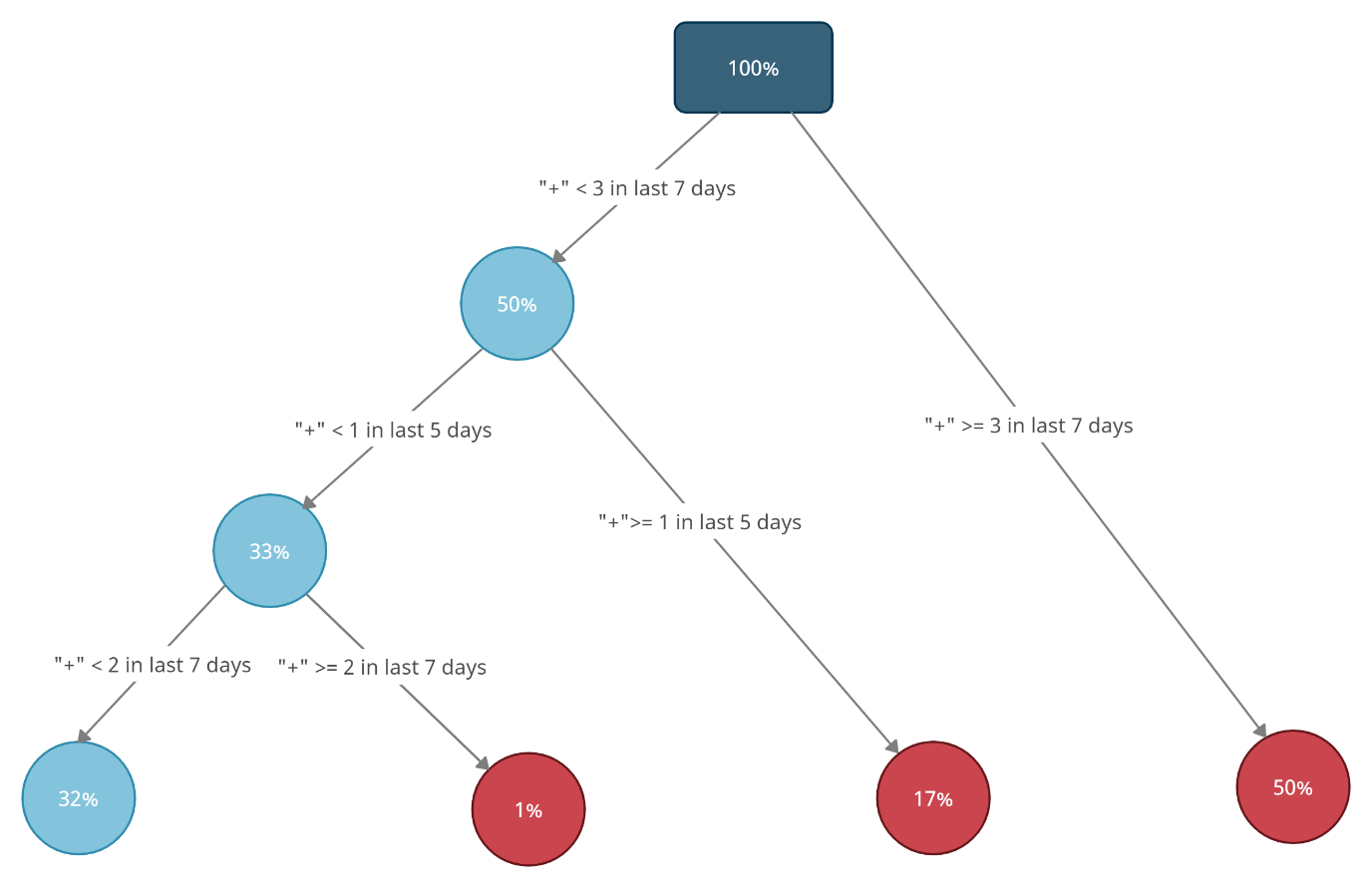


Figure S1. Classification tree trained with the training set using the outcome defined by 6-day time window. Wastewater time series features are used to predict individual COVID-19 test results. The red node means a positive predicted outcome and the blue node means a negative predicted outcome. The value inside each node denotes the percentage of the total data records that falls in the category of the node.  “+” means number of positive wastewater results. For example: “+ < 3 in last 7 days” means there were less than (<) 3 days of positive wastewater results in the last 7 days of wastewater testing.

Fig. S2.
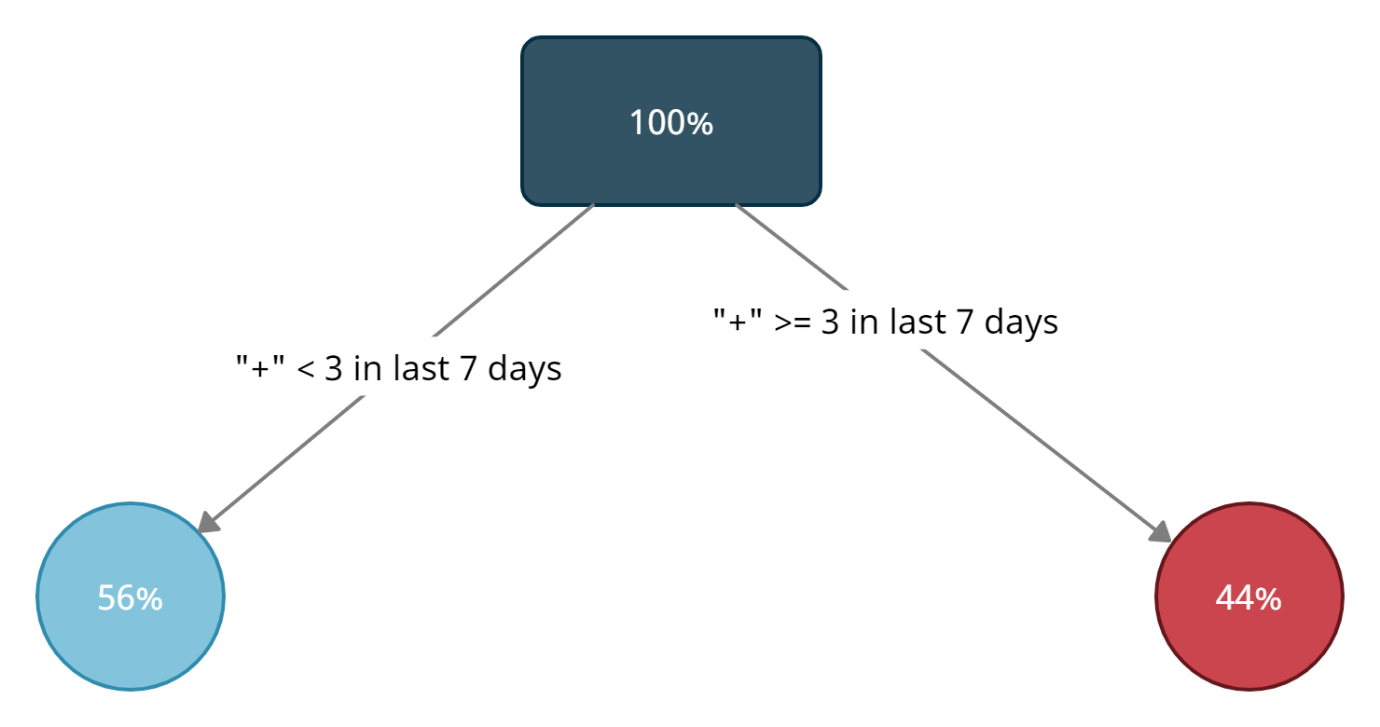


Figure S2. Classification tree trained with the training set using 1:1 relative weights for positive vs. negative outcomes. Wastewater time series features are used to predict individual COVID-19 test results. The red node means a positive predicted outcome and the blue node means a negative predicted outcome. The value inside each node denotes the percentage of the total data records that falls in the category of the node.  “+” means number of positive wastewater results. For example: “+ < 3 in last 7 days” means there were less than (<) 3 days of positive wastewater results in the last 7 days of wastewater testing.

Fig. S3.


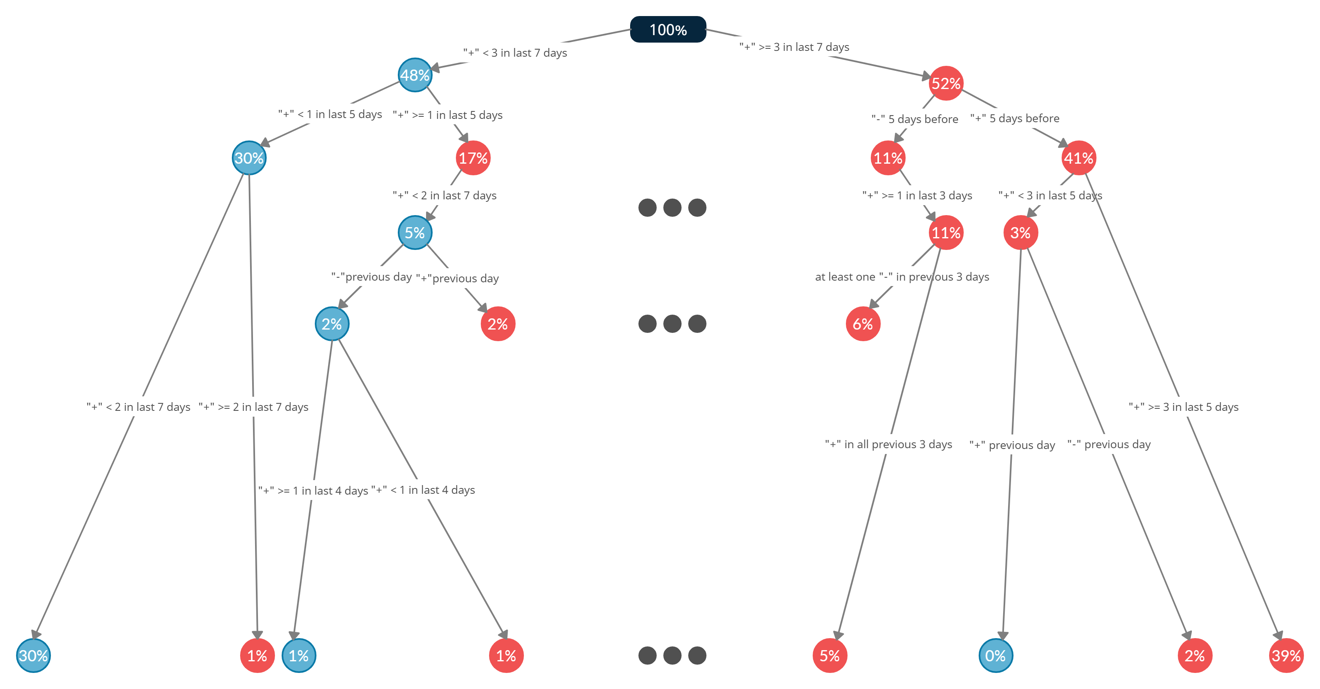


Figure S3. Classification tree trained with the training set using cp = 0.001. The red node means a positive predicted outcome and the blue node means a negative predicted outcome. The value inside each node denotes the percentage (rounded to nearest 1) of the total data records that falls in the category of the node.  “+” means number of positive wastewater results. For example: “+ < 3 in last 7 days” means there were less than (<) 3 days of positive wastewater results in the last 7 days of wastewater testing. As can be observed from the figure, the model is overly complex and produces categories that cover very small percentages (nearly 0%) of the data, which may lead to over-fitting and undermine the predictive utility of the model.

**Fig. S4.**


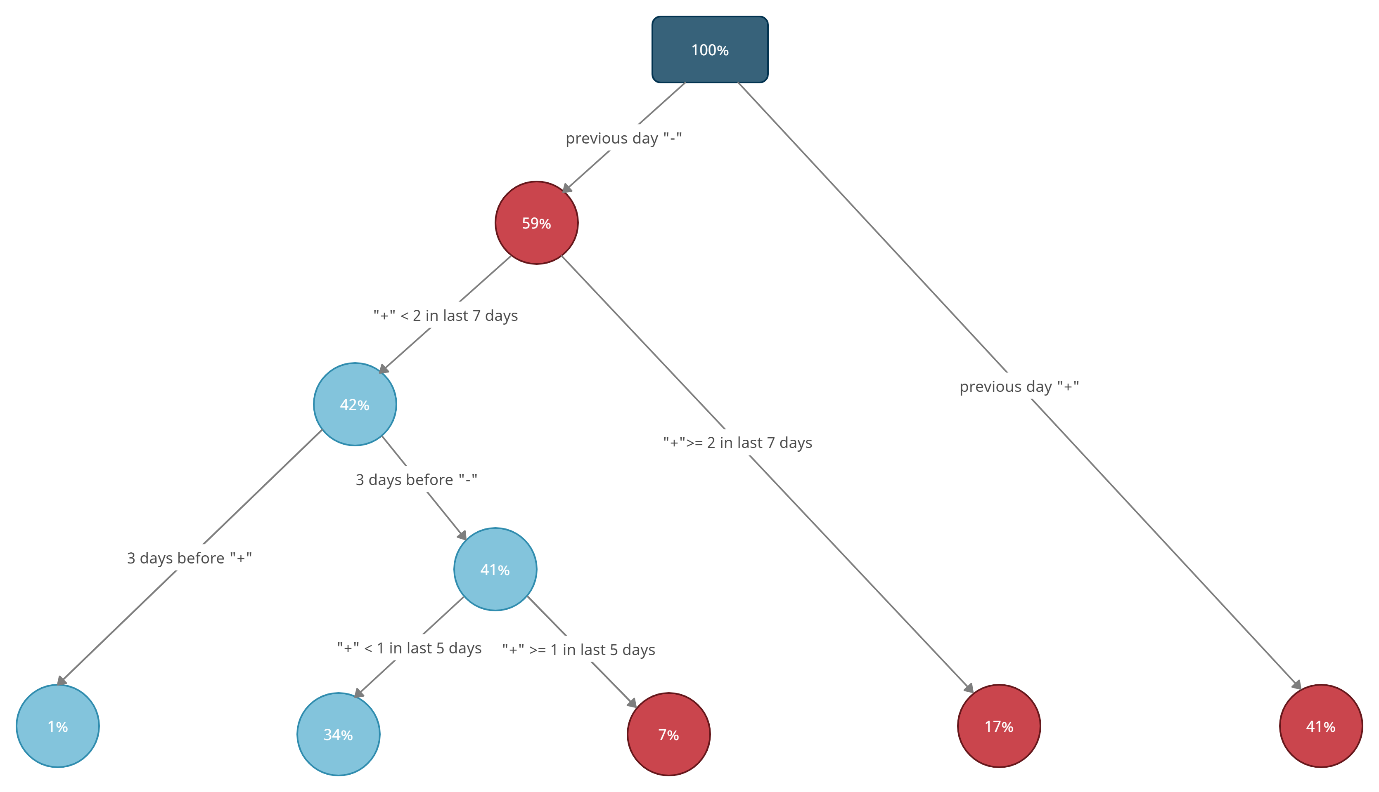


Figure S4. Classification tree trained with the testing set. Wastewater time series features are used to predict individual COVID-19 test results. The red node means a positive predicted outcome and the blue node means a negative predicted outcome. The value inside each node denotes the percentage of the total data records that falls in the category of the node.  “+” means number of positive wastewater results. For example: “+ < 3 in last 7 days” means there were less than (<) 3 days of positive wastewater results in the last 7 days of wastewater testing.

**Fig. S5.**


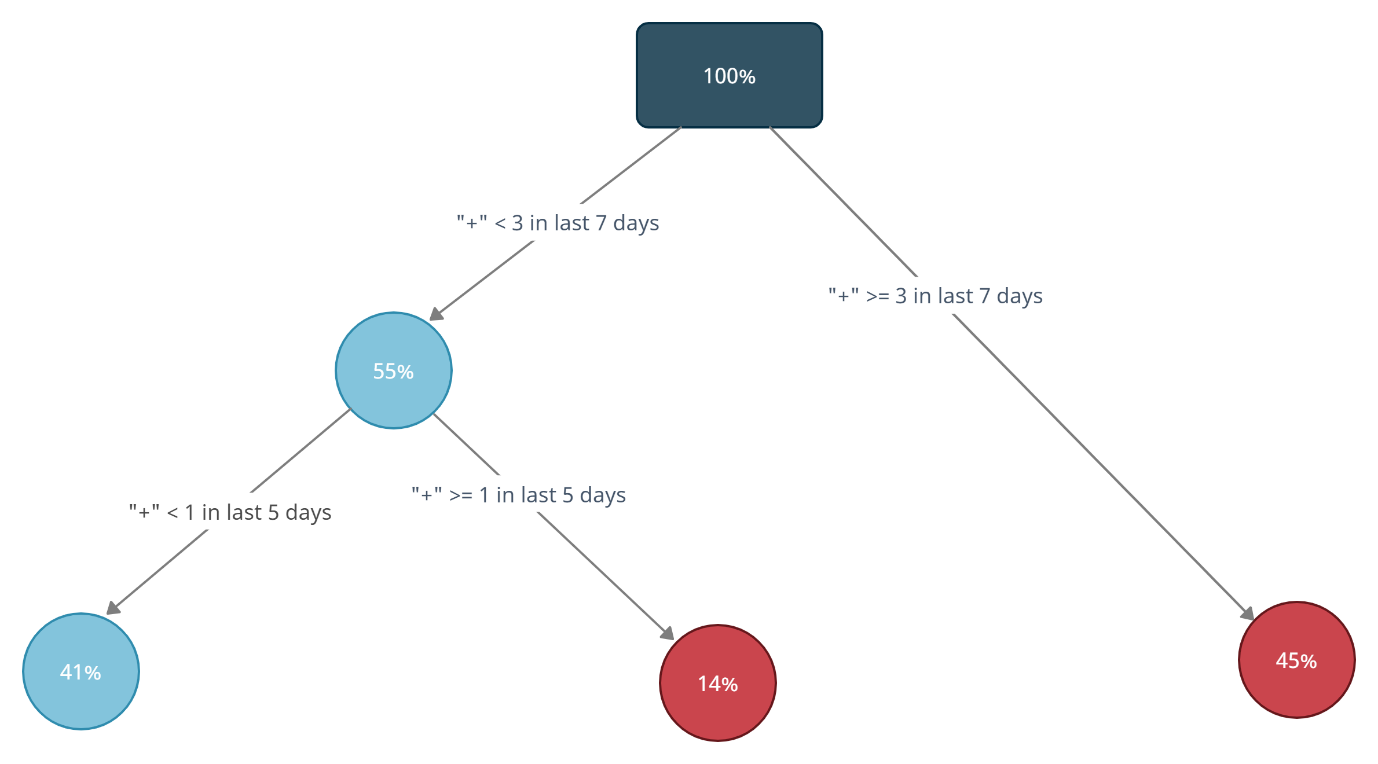


Figure S5. Classification tree trained with the training set including May and June data. Wastewater time series features are used to predict individual COVID-19 test results. The red node means a positive predicted outcome and the blue node means a negative predicted outcome. The value inside each node denotes the percentage of the total data records that falls in the category of the node.  “+” means number of positive wastewater results. For example: “+ < 3 in last 7 days” means there were less than (<) 3 days of positive wastewater results in the last 7 days of wastewater testing.

**Fig. S6.**


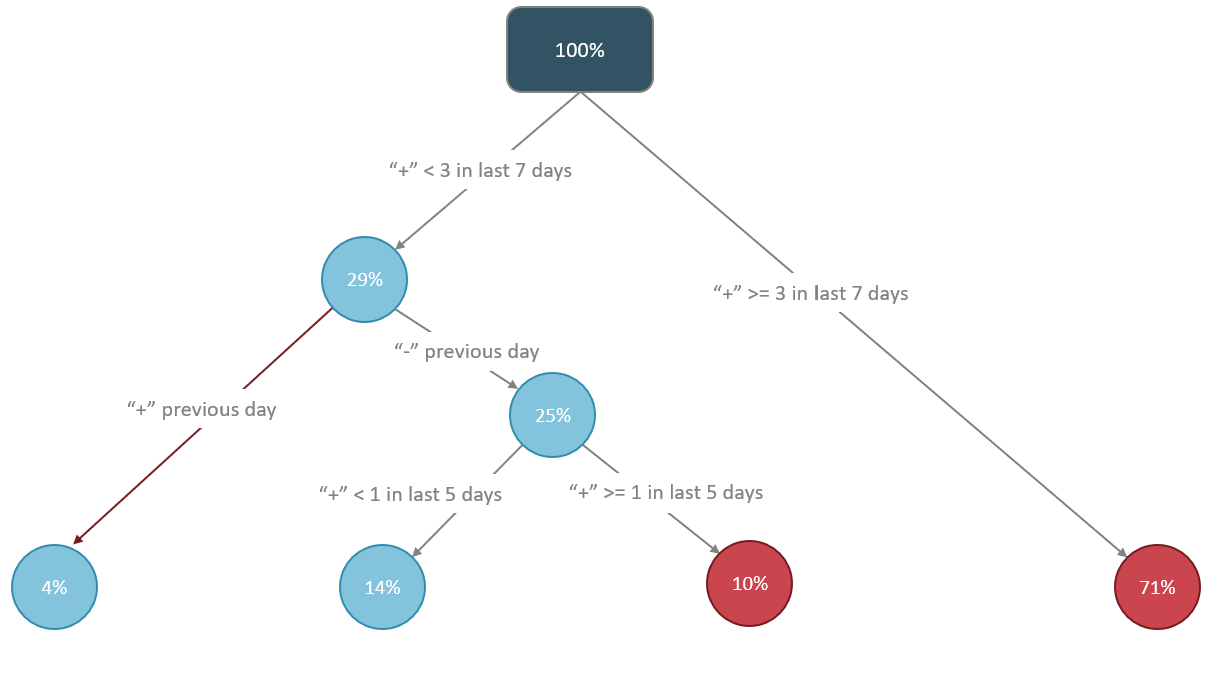


Figure S6. Classification tree trained with the training set using the data only from Fall 2020. Wastewater time series features are used to predict individual COVID-19 test results. The red node means a positive predicted outcome and the blue node means a negative predicted outcome. The value inside each node denotes the percentage of the total data records that fall in the category of the node.  “+” means number of positive wastewater results. For example: “+ < 3 in last 7 days” means there were less than (<) 3 days of positive wastewater results in the last 7 days of wastewater testing.

**Fig. S7.**

**
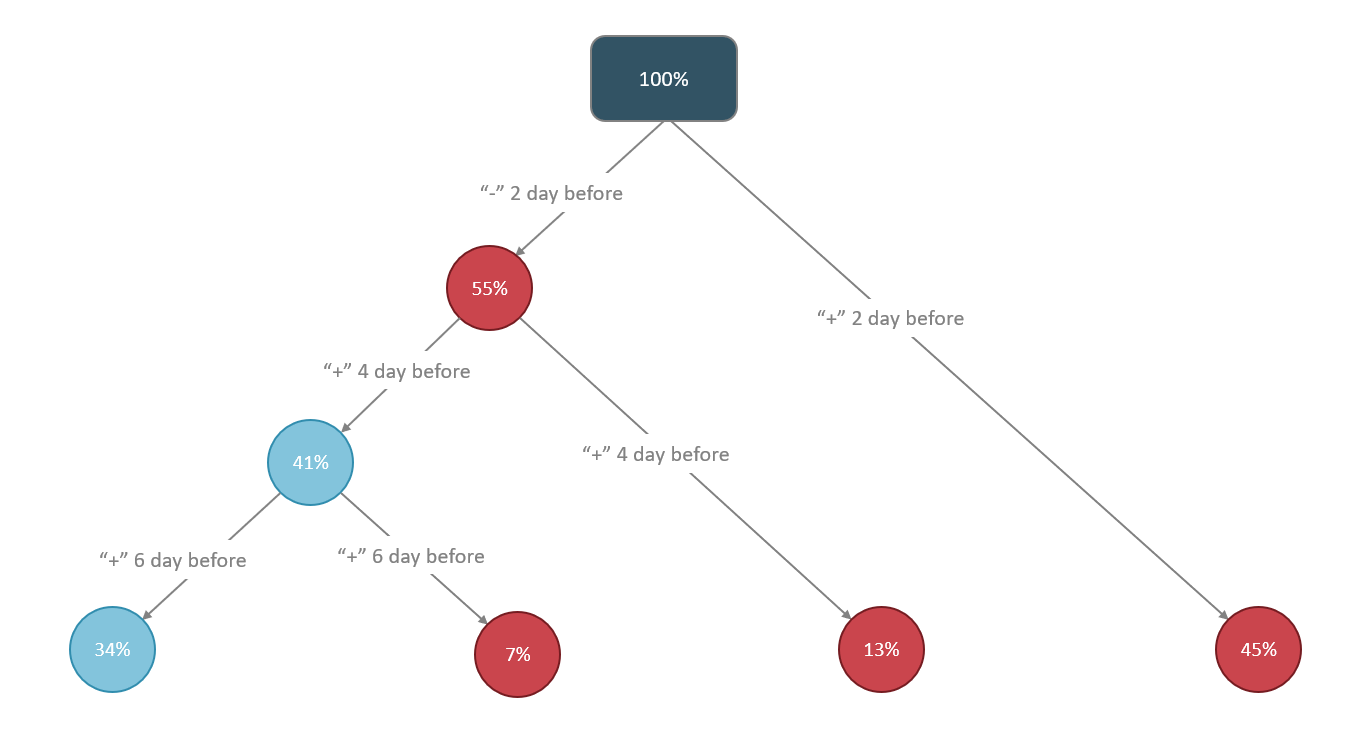
**

Figure S7. Classification tree trained with wastewater sampled every two days. Wastewater time series features are used to predict individual COVID-19 test results. The red node means a positive predicted outcome and the blue node means a negative predicted outcome. The value inside each node denotes the percentage of the total data records that fall in the category of the node.  “+” means number of positive wastewater results. For example: “+ < 3 in last 7 days” means there were less than (<) 3 days of positive wastewater results in the last 7 days of wastewater testing.

Table S1. Confusion matrix of results obtained from applying the tree model trained using the training set alone, with the outcome defined by 6-day time window, to the training set.

|  | Predict positive | Predict negative |
| --- | --- | --- |
| Actual positive | 81.6% | 18.4% |
| Actual negative | 41.4% | 58.6% |

Table S2. Confusion matrix of results obtained from applying the tree model trained using the training set alone, with the outcome defined by 6-day time window, to the testing set.

|  | Predict positive | Predict negative |
| --- | --- | --- |
| Actual positive | 75.0% | 25.0% |
| Actual negative | 38.0% | 62.0% |

Table S3. Confusion matrix of results from applying the model (trained with training set) to the training set, using 1:1 relative weights for positive vs. negative outcomes.

|  | Predict positive | Predict negative |
| --- | --- | --- |
| Actual positive | 68.1% | 31.9% |
| Actual negative | 20.4% | 79.6% |

Table S4. Confusion matrix of results from applying the tree model (trained with training set) to the testing set, using 1:1 relative weights for positive vs. negative outcomes.

|  | Predict positive | Predict negative |
| --- | --- | --- |
| Actual positive | 43.8% | 56.2% |
| Actual negative | 14.5% | 85.5% |

Table S5. Confusion matrix of results from applying the tree model (trained with training set) to the training set, using *cp*=0.001.

|  | Predict positive | Predict negative |
| --- | --- | --- |
| Actual positive | 84.4% | 15.6% |
| Actual negative | 34.4% | 65.6% |

**Table S6.** Confusion matrix of results from applying the tree model (trained with training set) to the testing set, using *cp*=0.001.

|  | Predict positive | Predict negative |
| --- | --- | --- |
| Actual positive | 64.6% | 35.4% |
| Actual negative | 29.7% | 70.3% |

Table S7. Confusion matrix of applying the tree model trained with the testing set only to the same testing set.

|  | Predict positive | Predict negative |
| --- | --- | --- |
| Actual positive | 79.1% | 20.8% |
| Actual negative | 35.6% | 64.4% |

Table S8. Confusion matrix of results of applying the tree model, re-trained with a new training set that includes data from May and June 2021, to the same new training set.

|  | Predict positive | Predict negative |
| --- | --- | --- |
| Actual positive | 74.8% | 25.2% |
| Actual negative | 27.1% | 72.9% |

Table S9. Confusion matrix of results from applying the tree model trained with training set using data from Fall 2020 to the training set.

|  | Predict positive | Predict negative |
| --- | --- | --- |
| Actual positive | 94.9% | 5.1% |
| Actual negative | 54.7% | 45.3% |

Table S10. Confusion matrix of results from applying the tree model trained with training set using data from Fall 2020 to the testing set.

|  | Predict positive | Predict negative |
| --- | --- | --- |
| Actual positive | 56.3% | 43.7% |
| Actual negative | 31.2% | 68.8% |

Table S11. Confusion matrix of results from applying the tree model (trained with training set) to the training set. The data is from sampling wastewater every 2 days.

|  | Predict positive | Predict negative |
| --- | --- | --- |
| Actual positive | 81.2% | 18.8% |
| Actual negative | 34.2% | 65.8% |

Table S12. Confusion matrix of results from applying the tree model (trained with training set) to the test set. The data is from sampling wastewater every 2 days.

|  | Predict positive | Predict negative |
| --- | --- | --- |
| Actual positive | 57.1% | 42.9% |
| Actual negative | 30.3% | 69.7% |

Table S13. Sensitivity, specificity and prediction accuracy when applying different machine learning models (trained with training set) to the training set by using the 2:1 weight ratio of positive vs. negative outcomes.

|  | Sensitivity | Specificity | Accuracy |
| --- | --- | --- | --- |
| Logistic regression | 83.0% | 64.3% | 76.7% |
| Logistic regression (with LASSO) | 83.7% | 58.5% | 75.3% |
| SVM | 80.0% | 62.4% | 74.1% |
| FNN | 84.4% | 65.4% | 78.1% |
| Random forests | 76.3% | 64.1% | 73.8% |

**Reference**

CDC. CDC Museum COVID-19 Timeline, accessed 10 Oct 2023. [https://www.cdc.gov/museum/timeline/covid19.html#](https://www.cdc.gov/museum/timeline/covid19.html)
